# Factors influencing H1N1 vaccine behavior among Manitoba Metis in Canada: a qualitative study

**DOI:** 10.1186/s12889-015-1482-2

**Published:** 2015-02-12

**Authors:** S Michelle Driedger, Ryan Maier, Chris Furgal, Cindy Jardine

**Affiliations:** Department of Community Health Sciences, University of Manitoba, S113-750 Bannatyne Avenue, Winnipeg, Canada; Indigenous Environment Studies Program, Trent University, 1600 West Bank Drive, Peterborough, Canada; School of Public Health, University of Alberta, 11405 – 87 Ave, Edmonton, Canada

**Keywords:** Infectious disease, Influenza, Immunization, Vaccination, Uptake, Perceptions, Aboriginal, Canada, Colonialism, Focus groups

## Abstract

**Background:**

During the first wave of the H1N1 influenza pandemic in 2009, Aboriginal populations in Canada experienced disproportionate rates of infection, particularly in the province of Manitoba. To protect those thought to be most at-risk, health authorities in Manitoba listed all Aboriginal people, including Metis, among those able to receive priority access to the novel vaccine when it first became available. Currently, no studies exist that have investigated the attitudes, influences, and vaccine behaviors among Aboriginal communities in Canada. This paper is the first to systematically connect vaccine behavior with the attitudes and beliefs that influenced Metis study participants’ H1N1 vaccine decision-making.

**Methods:**

Researchers held focus groups (n = 17) with Metis participants in urban, rural, and remote locations of Manitoba following the conclusion of the H1N1 pandemic. Participants were asked about their vaccination decisions and about the factors that influenced their decisions. Following data collection, responses were coded into the broad categories of a social-ecological model, nuanced by categories stemming from earlier research. Responses were then quantified to show the most influential factors in positively or negatively affecting the vaccine decision.

**Results:**

Media reporting, the influence of peer groups, and prioritization all had positive and negative influential effects on decision making. Whether vaccinated or not, the most negatively influential factors cited by participants were a lack of knowledge about the vaccine and the pandemic as well as concerns about vaccine safety. Risk of contracting H1N1 influenza was the biggest factor in positively influencing a vaccine decision, which in many cases trumped any co-existing negative influencers.

**Conclusions:**

Metis experiences of colonialism in Canada deeply affected their perceptions of the vaccine and pandemic, a context that health systems need to take into account when planning response activities in the future. Participants felt under-informed about most aspects of the vaccine and the pandemic, and many vaccine related misconceptions and fears existed. Recommendations include leveraging doctor-patient interactions as a site for sharing vaccine-related knowledge, as well as targeted, culturally-appropriate, and empowering public information strategies to supply reliable vaccine and pandemic information to potentially at-risk Aboriginal populations.

## Background

The emergence and rapid spread of an H1N1 influenza virus in the spring of 2009 prompted health systems around the world to support the development of a novel vaccine as part of their response strategies. In Canada, mass production challenges and initial limited availability forced health authorities to establish priority lists for vaccine roll-out by targeting those deemed potentially more susceptible or sensitive to infection or severe outcomes. In the Canadian province of Manitoba, a number of northern First Nation reserves experienced extraordinarily high rates of infection during the first wave of H1N1 (April 12 to August 29, 2009). This likely contributed to provincial health officials deciding to put all Aboriginal persons—First Nations, Metis, and Inuit—on the province’s H1N1 vaccine priority list [1,2]. Distribution of the new vaccine commenced October 26 during the second wave (August 30, 2009, to January 27, 2010) of the pandemic in Manitoba.

Being the first global pandemic in several decades, the H1N1 outbreak provides an opportunity to examine factors that influence uptake or refusal of a novel vaccine, and indeed this has been the focus of a growing body of literature [3,4]. However, studies looking at designated priority groups during the H1N1 pandemic have primarily focused on pregnant women, health care workers, or populations with high-risk co-morbidities [3-5]. The quantity of research that examines attitudes of particular ethnic groups towards novel vaccines (or even seasonal influenza vaccines) is quite limited [6-13], and an even greater gap exists for studies that investigate vaccine behaviors and related attitudes among Aboriginal communities in Canada. One study has shown that some First Nations and Metis individuals did feel apprehensive about being named as a priority group for the H1N1 vaccine [14]. This paper is the first to systematically document vaccine behavior with the attitudes and beliefs that influenced Metis people’s decisions to be vaccinated against H1N1 or not.

### The Metis people

Emerging out of mixed First Nation and European ancestry in the 17^th^ and 18^th^ centuries as a distinct political, social, and cultural national community, the Metis Nation is one of the three constitutionally recognized Aboriginal peoples who live in Canada [15]. Over the course of the 19^th^ century, this new nation established itself in the area where the Red and Assiniboine Rivers meet in what is now Manitoba. In this article, we will use the modern [16], unaccented form of the word “Metis,” in keeping with the form used by the current Manitoba Metis Federation (MMF – the Metis people’s official provincial representative organization) as a recognition of Manitoba as the birthplace of the Metis Nation [17]; outside Manitoba, the Métis are referenced in the accented form and we are consistent with that usage as relevant.

To better understand the vaccine-related attitudes and behaviors of the Metis participants in this study, it is necessary to give a brief sketch of the socio-historical context that came to influence contemporary decision-making processes. Since before and after Canadian confederation in 1867, Aboriginal populations’ claims of self-determination and territorial sovereignty were generally seen as obstacles to Canadian colonial ambitions of westward expansion and settlement [18,19]. Much like the experiences of other Aboriginal peoples, the Metis Nation has endured significant marginalization at the hands of the Canadian colonial-settler state. In the case of the Metis people, colonialism involved historical dispossession of lands through political manipulations and even outright war. Two key events illustrate this history. First, when Canada obtained Rupert’s Land from the Hudson’s Bay Company in 1869 – which contained the established areas of Metis settlement – the Metis leader Louis Riel negotiated the creation and entry of Manitoba into Canadian confederation. The terms agreed upon included provisions that were intended to protect and maintain Metis territorial integrity, as well as their political, social, and cultural life [20]. Those provisions never materialized, and as new waves of settlers were encouraged to take over what was intended to be Metis territories, many Metis people were forced to disperse into other areas of Manitoba and further west across the continental prairies [19]. Once more the Metis Nation attempted to establish a self-determined geo-political space for itself in what was to become the Canadian province of Saskatchewan in 1885. This time, the Canadian government sent in the army as a physical and symbolic show of force against what it perceived was a challenge to its own claims of sovereignty over the Canadian west [19,21]. The result was the defeat of the Metis forces at the Battle of Batoche after which Louis Riel was tried for treason and hanged [20]. In addition to these defining events in Metis history, the Metis people have also endured ongoing legacies of public and systemic racism and social exclusion [20,22-24]. Indeed it was only in 2013 that the Supreme Court of Canada found that following the creation of Manitoba, the Canadian government had failed to fulfill its constitutional obligations towards the Metis people [25]. Cumulatively, these factors can contribute to a sense of contemporary distrust towards any kind of government activity, even those that could otherwise be seen as more well-intended or potentially beneficial [26], such as a vaccination campaign for pandemic influenza.

Today, approximately 71,085 self-identified Metis people live diffusely in Manitoba’s cities, towns, villages, and unorganized territories, with approximately half living in the provincial capital city of Winnipeg [27]. Metis citizens in northern areas of the province generally live in rural, remote, and occasionally isolated communities – frequently adjacent to First Nation reserves. Collectively, Metis people in Manitoba have poorer health status compared to all other Manitobans, especially in the northern parts of the province as well as the downtown neighborhoods of Winnipeg [27]. Their lower health status is more clearly understood when viewed through the lens of the social determinants of health. As described by Reading and Wien [28], this approach holds that colonialism, social exclusion, racism, and denial of self-determination are all parts of historically distal, yet profound, determinants of health that created the conditions for the subsequent establishment of political, social, and economic inequities. Resulting disparities in these domains became further entrenched as more proximate and inter-related determinants have continued to exist to the present day. These include ongoing inequalities in access to health and education systems, continued experiences with racism and social exclusion, as well as limited community and environmental resources. The outcomes of such circumstances are realized in many contemporary peoples’ lives not only in poorer health status, but also lower socio-economic status, poorer living conditions, lower educational attainment, and lower health literacy [28-30]. These existing health and socio-economic disparities can potentially exacerbate and influence the disproportionate impact of a pandemic on a population [31]. Along with the proximity of many Metis in the province’s north to the hard-hit First Nation communities, these conditions all likely played a role in the province of Manitoba’s decision to include Metis on the H1N1 vaccine priority list during the pandemic.

### Measuring vaccine behavior and attitudes

Survey-based research has supplied information about many of the key factors in people’s pandemic vaccination decision-making processes: perception of disease risk, perceptions of vaccine risk and vaccine safety, established vaccine behavior, social discourses and environments, communication structures, knowledge of vaccines and influenza, and input from healthcare professionals [4,13,32-36]. Recent studies have also employed focus group methods, where participants can express their feelings, worries, and thoughts in their own words, thereby supplying deeper insight into these factors [5,37-39]. Focus group research with particular ethnic groups shows that factors reported in more general populations are mediated in different ways by different communities. For example, studies of novel vaccine acceptance among Canadian general populations indicate that concerns about vaccine safety play a role in influencing potential uptake [38], with particular issues being identified as: fear of potential side effects; feeling like laboratory animals; beliefs that the H1N1 vaccine could not have been tested sufficiently [37]. Studies of vaccine behavior among Latino and African-American populations in the United States (both of whom have had lower rates of uptake for seasonal and pandemic influenza vaccines) reveal that they have many of the same concerns. However, more complicated views also emerged from these populations in ways that amplified their concerns: misconceptions about vaccines were considerably widespread; many participants expressed a general lack of knowledge about vaccines; and they described negative historical and contemporary interactions with the government and health systems, and feelings of medical mistrust due to systemic racism (with African-Americans referencing the Tuskegee Experiments, and Latinos referencing recent laws that enable racial profiling in Arizona) [6,8-12]. Furthermore, there is also evidence that in communities that have had histories of racism and marginalization, the lower socio-economic status and lower educational attainment that is a consequence of those conditions can have a negative influence on health literacy and also the decision to vaccinate against influenza [12,30]. These and other recent studies confirm that threats to public health are indeed socially and culturally mediated, and when asked to take a novel vaccine, people can situate such requests within personal and community histories [10,40]. Thus, it is anticipated that the Metis participants in this investigation will have also experienced and interpreted the H1N1 pandemic in ways that are unique to their own socio-historical context, which may or may not share particular attributes with the communities examined in the studies noted above.

In a recent study focused on the vaccination behavior and attitudes of general population Canadians, Boerner and colleagues developed a framework to identify and categorize the multiple factors (listed and defined in Table 1) that influenced the uptake or refusal of the H1N1 vaccine during the pandemic [38]. Building on a social-ecological model (SEM) conceptualized by Kumar et al. [41], the framework facilitates analysis of the interplay of factors as they exist in intrapersonal, interpersonal, community, and system/policy domains [37]. As Boerner et al. show, the vaccine decision-making process is not always straightforward or simple, and often a variety of coalescing, competing, and at times contradictory, factors can all play a role. Some factors even have a dual function, acting both persuasively and dissuasively to influence decision-making. For example, media coverage positively and negatively influenced vaccine uptake by creating an atmosphere of palpable risk but also coming across to some members of the public as over-hyped [38]. These factors interact and form combinations that can be unique for each individual, but can also throw broader trends into sharper relief when viewed in aggregate. This study will further test the application of the SEM-informed framework, as employed by Boerner and colleagues, to see whether it can adequately account for the variety of experiences reported by Metis following the H1N1 pandemic in Manitoba, Canada.

**Table 1:** Components of a social-ecological model*

**Categories**	**Factors**	**Definitions**
System and institutional level factors	Vaccine roll-out and availability	Vaccine services and availability of H1N1 vaccine (e.g. how vaccine was delivered)
	Government communication	How and when H1N1 information from authorities was received and who delivered that information
	Institutional prevention activities	H1N1 prevention programs (e.g. vaccine clinics), provision of prevention information (e.g. information materials) instituted by an organization (e.g. school, workplace, etc.)
	Organization of the public into priority groups	Who was able to receive vaccinations and who was considered at-risk for contracting H1N1
Social context factors	Public discourse	How media covered H1N1, and how reliable or important media coverage was in relation to vaccination decisions
	“Bandwagoning”	Deciding to be vaccinated or not to be vaccinated because “everyone is doing it”
Interpersonal level	Interpersonal influences	Broad social pressure about what is expected of individuals by their social environment. Interaction with friends, family, coworkers, and others more generally in relation to vaccination and/or H1N1
	Interface with health professionals	Any mention of interaction and/or communication (or lack thereof) with a health professional
Intrapersonal factors	Habitual behavior	What individuals usually do or perceive in relation to the seasonal influenza or other vaccinations
	Altruism	An individual’s decision to vaccinate or not to vaccinate is made in order to protect or benefit someone else or to forego vaccination when vaccine supply is low in order to allow those more at risk to vaccinate
	“Free-loading”	Relying on herd immunity to protect against H1N1 and therefore deciding not to be vaccinated
	Vaccine risk perception	How safe or unsafe individuals felt the H1N1 vaccine to be
	Personal risk perception	How at-risk individuals perceived themselves to be in contracting and becoming seriously ill from H1N1
	Knowledge state	Knowledge or lack of knowledge regarding H1N1, vaccination, vaccination roll-out process, priority groups
	Trust	Who is trusted and not trusted has an influence on what information one accepts and subsequent actions
	Protected values	Ideals held so strongly that individuals would be unwilling to act counter to these values no matter what the benefits might be
	Past experiences	Past experience with vaccines and/or influenza
	Perceived alternatives	Tendency to prefer natural products and substances or other non-medical alternatives to vaccination (such as eating properly and exercising)

*As adapted by Boerner et al. [38].

## Methods

**Figure 1:**
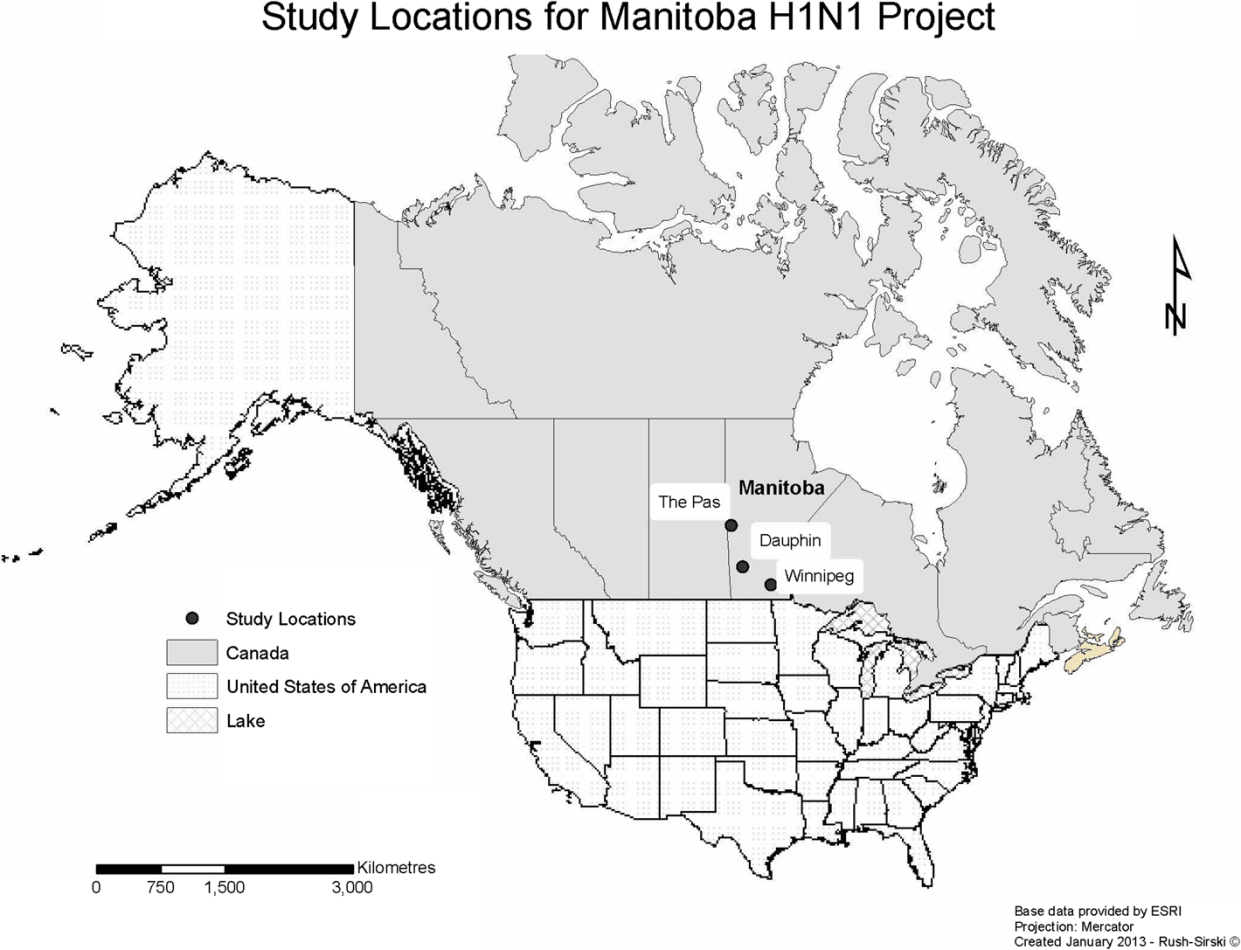
**Study locations for Manitoba H1N1 project.** Aside from Winnipeg, the other two study locations show the nearest urban centres and are thus regional approximations and not actual study sites. The names of the rural and remote communities that were visited are not listed in order to maintain participants’ confidentiality.

This study is part of a larger research project that examined risk and trust in decision-maker actions in First Nations, Inuit, and Metis contexts [42,43]. The respective Metis component of the project focused on general perceptions of governments’ overall response to the H1N1 pandemic, with vaccination decision-making forming only one specific part of the investigation. Data were obtained using 17 focus groups with Metis individuals in four communities in Manitoba. Eight focus groups were held in the urban centre of Winnipeg, four were held in a rural community in a western region of Manitoba, and five were held in two remote communities in two northern regions of the province. See Figure 1 for study locations, although in order to protect the confidentiality of rural and remote community members only the nearest urban centres are shown. The majority of focus groups took place in 2010 (n = 15) with the final two held in 2013 and only in the western rural community. These two later focus groups included 11 participants who took part in the initial focus groups in 2010, plus 9 new participants. Although returning to this community was prompted by questions relating to the more general project, re-visiting allowed for further data collection on H1N1 vaccination decisions with new participants, plus an opportunity for community members to co-interpret preliminary conclusions and results, and provide valuable clarification and further insights. All focus groups were audio-recorded, transcribed, audio-verified for accuracy and were carried out by the principle investigator and a research associate who also recorded field notes throughout the process [44-46]. The principle investigator is Metis, while the research associate has an MA in Native Studies with experience conducting focus groups in aboriginal communities. Research ethics protocols were approved by University of Manitoba Health Research Ethics Board (Reference number: H2010:008).

This study followed principles of community-based research that promotes close collaboration between the investigators and the involved communities [47]. The Manitoba Metis Federation (MMF) played key roles throughout the research process as community collaborators – from research design and knowledge dissemination, to ensuring that cultural protocols were followed throughout the project. MMF staff also facilitated recruitment by placing posters in strategic locations within communities along with word of mouth advertising. Participants provided written or oral consent for their participation and received a $50 honorarium for their time. The focus groups were premised on the basis of collegiality and mutual respect and involved the sharing of a communal meal (prepared by a community member in the rural/remote focus groups who was compensated for their time and skill).

Participants completed a demographic questionnaire and a survey that asked whether or not they were vaccinated during the H1N1 pandemic. Focus group participants were then asked a series of questions designed to get their perspectives on how they perceived the pandemic, where they got their information, and how they made their vaccination decisions. Once the discussions were transcribed and audio-verified, data were imported into NVivo9™ for analysis. Researchers then coded the data into the SEM framework and its constituent categories refined by Boerner et al. shown in Table 1 [38]. Data were then quantified by adding up the number of times particular factors were identified by participants, although if a participant mentioned the same factor multiple times during the conversations, it was counted only once. While quantifying qualitative data is typically discouraged in practice – because it detracts from the contextual information that qualitative research methods produce – it is done intentionally here. Specifically, counting participant responses helps to identify the most salient influencers as well as to gain insight into the interplay of factors in individuals’ decision-making that ultimately tipped the balance for or against receipt of the vaccine.

## Results

### Demographic characteristics and survey results

A total of 128 people participated in the focus groups, 56 in Winnipeg and 72 in rural and remote communities. Demographic information for the focus group participants is displayed in Table 2. Overall, there were more female participants (66%) who took part in the discussions. The level of educational attainment among participants echoes official Canadian statistics showing disparities between Aboriginal and non-Aboriginal populations. Of note, 47% of all participants did not have a high-school diploma, compared to 2006 Census figures that show that 11% of all Manitoba residents (aged 25–64 years) did not have a high school diploma [48]. According to the same Census data, 22% of all Manitoba residents hold a university/college degree, diploma, or certificate [48], whereas only 5% of Metis participants in this study held a university/college degree. At the same time, household incomes levels for this participant cohort were substantially lower than Manitoba (all residents) averages: 79% of Metis study participants live in households with an income of $50,000 or less, whereas provincial statistics show that in 2010 the average household income for all Manitoba residents was approximately $66,530 [49].

**Table 2:** **Metis focus group participant characteristics**

	**Overall %**	**Winnipeg**	**Rural/Remote**
	**(n = 128)**	**(n = 56)**	**(n = 72)**
**Gender**			
Men	(n = 44) 34%	(n = 17) 30%	(n = 27) 38%
Women	(n = 84) 66%	(n = 39) 70%	(n = 45) 63%
**Age (years)**			
18-34	(n = 34) 27%	(n = 21) 38%	(n = 13) 18%
35-54	(n = 37) 30%	(n = 15) 27%	(n = 22) 31%
55+	(n = 57) 45%	(n = 20) 36%	(n = 37) 51%
Education*			
Less than Grade 5	(n = 10) 8%	(n = 5) 9%	(n = 5) 7%
Grade 5-10	(n = 50) 39%	(n = 17) 30%	(n = 33) 46%
Grade 11-12	(n = 45) 35%	(n = 19) 34%	(n = 26) 36%
Some University/College	(n = 9) 7%	(n = 5) 9%	(n = 4) 6%
University or College Degree	(n = 5) 4%	(n = 4) 7%	(n = 1) 1%
Postgraduate Degree	(n = 1) 1%	(n = 1) 2%	(n = 0)
Household Income (Can $)*			
0 – 20 000	(n = 69) 54%	(n = 30) 54%	(n = 39) 54%
20 001 – 50 000	(n = 32) 25%	(n = 14) 25%	(n = 18) 25%
50 001 – 80 000	(n = 4) 3%	(n = 1) 2%	(n = 3) 4%
80 001+	(n = 2) 2%	(n = 1) 2%	(n = 3) 4%
2009 H1N1 Vaccine (yes)**	(n = 72) 56%	(n = 26) 46%	(n = 46) 64%
2010 Seasonal Flu Vaccine (yes)***	(n = 46) 39%	(n = 18) 32%	(n = 28) 44%

*21 Participants out of all focus groups only partially filled out demographic questionnaires. All data received is shown here.

**2009 H1N1 vaccination status is only known for 126 participants.

***This total only includes responses of the 119 participants who took part in the 2010 focus groups. Of the 9 new participants who took part in the 2 co-interpretive focus groups in 2013, 3 said that they intended to receive the 2013 seasonal flu vaccine. The remaining 6 said they did not intend to receive it. The other 11 participants in the co-interpretive 2013 focus groups were present in 2010 and their responses are included in the 2010 Seasonal flu vaccine totals. The 39% vaccination rate for 2010 seasonal flu vaccine is thus out of a denominator of 119.

Seventy-two participants (56%) indicated that they received the H1N1 vaccine. There was considerable geographic variability in the rates of vaccination among participants. Rural and remote communities had a considerably higher proportion of vaccination (64%). Although rural and remote are aggregated together in Table 2, the highest rate of vaccination was in the two northern remote communities, where 39 out of 47 participants (83%) were vaccinated. In the focus groups held in the rural community in the western region of the province, only 7 out of 24 participants (29%) received the vaccine. In the urban center of Winnipeg, 26 out of 56 participants (46%) were vaccinated.

### Factors influencing H1N1 vaccination

Even among this discrete population there was considerable variation in decision-making processes. While many participants listed a variety of persuasive or dissuasive factors in their decision making, some named only one or two influences, and a few preferred not to share their rationale for their decision – a decision the research team respected, knowing that those participants attended to share their thoughts on the broader research project topics that were also part of the focus group discussions. Sometimes several factors mutually supported a decision for a participant, and sometimes persuasive and dissuasive factors existed simultaneously and in conflict. In some instances, participants reported that they had been more inclined to vaccinate at one time, and less at another, in response to particular positive/negative influences as the pandemic evolved. Nevertheless, all relevant factors identified by each participant were included in the data in order to portray the broad spectrum of responses, their interplay, and which ones were found to be most common among all participants. The SEM-informed framework with the populated data is found in Table 3. Results are organized here according to the four macro-categories of the SEM, with the most salient constituent factors being highlighted in each. Representative comments from participants are also included to add voice to the data.

**Table 3:** Reported factors in H1N1 vaccine decision-making*

**Factors**	**Participants who reported vaccinating against H1N1 (n = 72)**		**Participants who reported NOT vaccinating against H1N1 (n = 54)**	
	**Winnipeg**	**Rural/Remote**	**Winnipeg**	**Rural/Remote**
	**(n = 26)**	**(n = 46)**	**(n = 30)**	**(n = 24)**
**System/Institutional level**				
Definition of priority groups	8(7+/−1)	7(5+/2-)	6(3+/3-)	5(2+/3-)
Government communication	13(11+/2-)	6+	11(6+/5-)	0
Vaccine roll-out and availability	6+	1+	2(1+/1-)	2(1+/1-)
Institutional interventions	2+	0	0	0
**Social Context level**				
Media coverage	10(9+/1)	13(7+/6-)	11(1+/10-)	6(1+/5-)
“Bandwagoning”	7+	6+	0	0
**Interpersonal level**				
Interpersonal influence	11(7+/4-)	13+	14(3+/11-)	5(3+/2-)
Interaction with health professionals	10(9+/1-)	4+	0	2(1+/1-)
**Intrapersonal level**				
Habitual behavior	7(4+/3-)	5(3+/2-)	6(2+/4-)	8(2+/6-)
Altruism	4+	3+	0	0
“Free-loading”	0	0	0	0
Vaccine risk perception	12-	19(1+/18-)	18-	17-
Personal risk perception	15(14+/1-)	17(15+/2-)	17(5+/12-)	8(2+/6-)
Knowledge state	13(5+/8-)	17(1+/16-)	25(1+/24-)	12-
Trust	7(5+/2-)	1-	5-	5-
Protected values	1-	0	6-	4-
Past experience	6(4+/2-)	5(3+/2-)	5-	7(2+/5-)
Perceived alternatives	0	2-	8-	5-

*The non-bracketed value in each cell represents the total number of participants who reported the factor. The bracketed values followed by “+” reflect instances where that factor positively influenced a decision to vaccinate; whereas bracketed values followed by “-” reflect instances where that factor negatively influenced a decision to vaccinate. In either case, there were instances when participants reported that they acted contrary to the way a factor influenced them.

### System/environmental or institutional factors

System/environmental or institutional factors refer to activities of health authorities, including the dissemination of pandemic or vaccine related information, as well as actual vaccine distribution activities. Government communication activities that included generalized recommendations promoting vaccination to protect from H1N1 infection did positively influence a number of participants (n = 17) to get vaccinated. Prioritizing Aboriginal people to be among the first to receive the vaccine had both a positive and negative influence on participants’ vaccination decisions, and was listed by some as one of the reasons why they got the vaccine (n = 12) and why they did not (n = 6). On the one hand, vaccine recipients occasionally noted that the prioritization played a key part in making the decision to get vaccinated. “*Yes, I was vaccinated in 2009 because it was a big deal. Everyone was saying you have to go get it or else you’re going to die especially if you’re Aboriginal*.” On the other hand, those who did not receive the vaccine indicated that they believed that through prioritization the government was testing the vaccine on Aboriginal people before making it available to the general population and avoided it accordingly. One sentiment, shared by a number of others, was expressed this way: “*Why did they pick on the Metis to have that shot first? Like that’s what I wanna know.*” As is discussed further below, the concern over being treated like “*guinea pigs*” was shared by vaccinated and non-vaccinated Metis participants alike.

### Social context

When participants described getting information from the media, they would most often indicate that they heard or saw something by using generalized terms, such as “*on the news”* (typically television), “*in the paper*,” and “*on the internet*” (a news website or a blog). Or, they would mention a particular website (eg. Youtube), but references to particular social media forums, such as Facebook or Twitter, were rare, as they were only emerging or relatively new at the time of the pandemic. In any case, media coverage of the pandemic and the vaccine had starkly divergent influences on participants’ vaccination decisions. Some of those who got the vaccine (n = 16) mentioned that the television media had instilled a generalized feeling of panic and fear that prompted them to get vaccinated. One person who said they had never previously received a seasonal influenza shot, but had received the H1N1 vaccine, noted, “*I think the media played a big role in putting a scare into a lot people.*” Various media sources were also named by many participants (n = 15) as contributing to their decision not to vaccinate. Some felt that news media over-sensationalized the severity of the pandemic, while others did not get vaccinated because certain television media or internet sites had informed them that the vaccine had mercury in it or that the vaccine could have side effects, even causing some deaths. “*On the news some people got them and gone and died from it. They got sick and they were dying. So I didn’t want to take that chance. I didn’t get the needle.*” ‘Bandwagoning’, either from a sense that all those around them were being vaccinated, or feeling compelled to get the vaccine after seeing line-ups at clinics, also figured as a prominent persuasive factor (n = 13).

### Interpersonal factors

Participants’ families and friends equally played a prominent role in influencing vaccination decisions for many of the Metis participants (n = 43). Many indicated that, despite not wanting to get the H1N1 vaccination for a different reason, often the prodding of a child or a parent was enough to overcome their initial apprehension. As one participant noted, “*I mean I probably wouldn’t have got a shot, but it was my daughter that insisted.*” Conversely, many who did not get the vaccine (n = 13), as well as some who did (n = 4), also mentioned that friends or family had urged them not to get the H1N1 vaccine, citing concerns about vaccine safety, misconceptions about vaccines, or anecdotal stories about what happened to them or someone else. “*People were saying, ‘oh you should or you shouldn’t and you might be more susceptible to getting more sick or it could make you sick.’ So I just chose not to.*” Some participants (n = 6) chose not to get vaccinated despite having family or friends who encouraged them to get vaccinated.

Recommendations from a health professional were also referenced (n = 13) as a positive influencer in getting the H1N1 vaccine. In fact, in almost every instance when a participant mentioned that a health professional had advised them to get the vaccine, that individual was vaccinated. Some explicitly expressed that they held great trust in their doctor’s advice, with a few participants even mentioning that the advice of a doctor over-rode an existing desire to not be vaccinated against H1N1. “*Based on my decision I wouldn’t have gotten it. The reason I got it was everyone was getting it, the doctor said, you know, I really think you should get it.*” In one instance someone was discouraged by their doctor from getting the vaccine because they were pregnant, but eventually got it due to pressure from her community and learning about it through their own research and reading. The only other time within our dataset that a doctor said to a participant that they may not need to have the vaccine was when that person may have already had the H1N1 virus.

### Intra-personal factors

While Boerner and colleagues [38] identified 10 factors grouped into the category of intra-personal factors, only nine were expressed during these focus groups. No participants alluded to an influence of ‘free-loading.’ Among the remaining factors, the three that were mentioned the most in participants’ decision-making processes were their perceptions of risk relative to their knowledge about the vaccine and the pandemic (n = 67), to vaccine safety (n = 66), and finally to the pandemic threat itself (n = 57). There also emerged a close relationship between people’s knowledge deficits and their perceptions of vaccine safety.

Vaccinated or not, most participants who referenced informational factors felt that they had a significant lack of information about the H1N1 vaccine, or vaccines and pandemics in general. Participants did not feel that they received enough information about the “*pros and cons*” of the H1N1 vaccine. Many participants wanted more information on how vaccines work and the pandemic itself, not just instructions telling them to go and get vaccinated. Faced with such a self-acknowledged information gap, or so much seemingly contradictory information, some participants believed that governments or health authorities were withholding information about the pandemic, suppressing anything negative about the vaccine, or only communicating potentially negative aspects about the vaccine after most people had been vaccinated. “*They give you a little bit of information but then after you get the needle after they’re gone, they said other stuff after but it’s too late. You’ve already got your needle. They don’t tell you until about two weeks after what the risks were, you could get side effects, if you get this. I can’t rewind.*” The dissuasive influence of knowledge gaps was most commonly referenced by those who refused the vaccine (n = 36). However, a lack of knowledge about the vaccine or the pandemic was reported a considerable number of times by those who received the vaccine (n = 24), stimulating degrees of fear and anxiety among them. As another participant noted: “*Now you put something into us that we don’t even know what the hell that is.*”

Closely tied to the issue of knowledge deficits, concerns related to the safety of the novel H1N1 vaccine were extremely common in the participants’ discussions. Safety concerns frequently reported by both those who were vaccinated (n = 30) and by those who chose otherwise (n = 35). Most commonly, participants expressed anxieties about potentially harmful ingredients, insufficient testing, or fear of severe or long term side effects of the vaccine. For some participants who received the vaccine, such concerns over vaccine safety caused them to regret their decision, leading them to say that they would not take such a risk again in the future. As one participant noted: “*I did it not by my choice but everybody else’s choice, like the doctors. If I have my way now, I’m not going to get it*.”

In these focus groups, concerns over a perceived lack of vaccine testing and of feeling like a *“guinea pig*” were explicitly stated. Coupled with concerns over vaccine safety, these factors deterred many from getting the vaccine, and added a palpable sense of fear and vulnerability to those that did. Participants frequently framed their anxiety over vaccine safety within an extant colonialism, and this became especially the case when such concerns were mentioned in combination with the prioritization of Aboriginal people for the vaccine. Such sentiment came out regardless of urban or rural/remote location. As a participant in a Winnipeg focus group said: “*Something about it just didn’t seem right…especially when they started gearing towards Aboriginal people, right away I thought they’re just trying to use us, like she said, like guinea pigs. Like, let’s push it on them now. It made me think even more*.” Another Winnipeg participant said: “*They got no use for us so they’re going to toy with us, you know. And try and kill us, H1N1*,” to which another participant who received the vaccine agreed: “*I was thinking of that, you know how they wiped out nations before.*” Similarly, after a participant from a remote community explained that she thought the pandemic and the vaccine were part of a plot to eliminate Aboriginal people, she noted: “*Honestly, that’s why I didn’t get the needle…I just didn’t trust the government*.” And as another remote community member who was vaccinated expressed: “*They could have just been using us as guinea pigs…there’s such a thing as chemical warfare.*” Upon finding out that the 2010 seasonal influenza vaccine included the pandemic H1N1 influenza A strain, some participants also said they would refuse it, or expressed regret or anger if they had already received it. Only a small number of participants (n = 7) who received the H1N1 vaccine contextualized their decision with their annual routine of getting a seasonal influenza vaccination and as such did not express any anxiety about the safety of the H1N1 vaccine.

Perceived risk from the pandemic corresponded strongly to the participants’ vaccination status. Many participants who were vaccinated against H1N1 indicated that the threat from the pandemic was a major motivating factor in their vaccination decision (n = 29). Especially in the northern remote communities, participants described an atmosphere of tense fear during the pandemic, and many connected that feeling to their vaccination decision. In most instances, anxiety about the potential severity of the pandemic, often in tandem with other influences such as advice from doctors, peer influence, among others, was enough to over-ride existing fears about vaccine safety. Nevertheless, the tension between the risks of the pandemic and the vaccine remained dynamic and persistent, as summed up by one participant who received the vaccine: “*For me, being pregnant, like with the risks of the actual flu and they were pretty scary. And then also with the risks, like reading over when I had to sign a consent form. Reading the risks after getting the shot, that was kind of scary. But then after talking it over with the nurses and doctor and they kind of convinced me to get the shot. And then there are things I didn’t like and I was really worried about after getting it.*” Conversely, for those who were not vaccinated, many (n = 18) did not feel particularly threatened by the pandemic and therefore did not feel the need to be vaccinated. These participants often referenced alternative methods of avoiding infection, such as personal sanitation behaviors: “*I think that if you keep yourself clean, wash your hands, stuff like that, like if I do that, I figure I’ll be okay.*” Or, they were generally indifferent to the pandemic, reporting that “*it wasn’t an issue for me*,” or that they believed it was no different than seasonal influenza: “*Everybody knows there’s always flu viruses*.”

## Discussion

When compared to Boerner et al.’s study of general population Canadians (n = 130), a number of the factors influenced Metis participants’ attitudes in very similar ways. Pandemic risk perception, interactions with peers and healthcare providers, and media coverage all positively influenced decisions to vaccinate [38]. Most notably, in both studies pandemic risk perception was the most commonly cited factor in positively influencing vaccination, substantiating other research that has associated the likelihood of vaccination with perceived risk of illness [50,51]. However, this study finds that the threat of the pandemic was likely socially amplified in a unique way for its Metis participants [52]. Discussions showed that pandemic risk was experienced and felt collectively by many Metis, and especially in the remote northern communities that were visited. In those smaller and more culturally homogenous communities, risk of the pandemic was seen as especially high—a social and geographical context that has also been associated with higher rates of vaccination in other indigenous communities [7]. In Winnipeg, vaccinated Metis participants likewise expressed concerns about a high risk from the pandemic (n = 14), but conversely many of those who were not vaccinated did not feel at risk (n = 12). This could be explained by the fact that Winnipeg’s Metis population is much more diffuse and less homogenous, and media reports concentrating on northern communities may have led some to believe the risk was more confined to the those areas [53]. Moreover, none of the non-vaccinated Winnipeg participants (n = 30) referenced the influence of a healthcare provider or professional in their decision, which may signal the existence of potential unidentified barriers to health care access in an environment where such access should ostensibly be easy.

The strong ties between a healthcare professional’s recommendation and vaccination status is also supported by other studies [3,54]. Certainly, health professionals can play a valuable role in vaccine education and in dispelling common misconceptions as doctors are often ascribed a high level of trust and credibility. However, healthcare professionals have also been shown to ‘fall short’ in providing enough information about vaccines to indigenous populations [7]. Plus, as will be discussed more fully below, leveraging the potential trust of the doctor-patient relationship is not so straightforward when set against a history of negative interactions between Aboriginal people and Canadian health systems.

At the same time, vaccine risk perceptions, media, interpersonal interactions, and knowledge factors were all found to have potentially negative influences on vaccine decision-making in both Metis and Canadian general population focus groups [38], further verifying results found elsewhere [33,55]. That media influence is found on both positive and negative ends of the decision-making spectrum echoes Boerner et al.’s findings that point to particular dissonances in media-related risk messaging: the content can be effective at times, but can also become editorialized, fatiguing, or perceived by the public as conflicting or sensationalized [38,56]. The similar dual functioning of interpersonal influences is likely a partial downstream effect of those inconsistencies infiltrating discourse within social circles [38].

Of the key areas where notable differences existed between Metis and general population Canadians, the rate of vaccination is particularly noteworthy, with 56% of the Metis focus group participants reporting being vaccinated. Although not generalizable to all Metis in the province (for which there are no available data on province-wide Metis H1N1 vaccine uptake), it does bear a reasonably close resemblance to the official First Nations vaccination rate of 60% in Manitoba [2]. By contrast, the percentage of participants vaccinated in Boerner and colleague’s study of general population Canadian participants was 37% [38], which is similar to Canadian averages (ranging from 32% to 45% across all Canadian provinces) [38] and is identical to the overall vaccination rate for all Manitoba residents (37%) [2]. The reported high perception of pandemic risk reported by vaccinated Metis participant does offer some explanation for their relatively high rates. Additionally, in the two northern remote communities (where most participants were vaccinated) each had a community health centre on-site (albeit only lightly staffed with a community health worker and an occasional itinerant physician who provides a limited number of services) where residents could receive the H1N1 vaccine with relative ease. In the rural community where the proportion of vaccinated participants was quite low (7 out of 24), the participants lived generally more spread out over a larger territory in a rural municipality, and not in a discrete, closely proximate, culturally homogenous community with a centrally located health centre close to people’s homes. Also, all but one participant was over 55 years of age, pointing to a substantially older demographic residing in the region. Here the most common factors reported were concerns over vaccine safety and strict aversions (habitually or value-based) to vaccines – H1N1 and generally – plus their stated lack of knowledge about the vaccine and the pandemic.

Interestingly, routine behavior relative to seasonal influenza vaccinations was not as prominent of a predictor for getting the H1N1 vaccine among Metis participants as it was for general population Canadians [38] and for populations elsewhere [34,54]. However, the most noteworthy differences from general population Canadians in Boerner and colleagues’ study were Metis participants’ high reportage of perceived risk from the vaccine as well as knowledge deficits, regardless of their vaccination status. Of course, for those who chose to be vaccinated, ultimately another positively-influencing factor (threat of the pandemic, etc.) sufficiently over-rode any negative influencers that existed at the time – a decision-making process that has also been found elsewhere in the literature [8] and which further illustrates of complex interplay of the factors at play.

Studies by Boerner and colleagues [38] and Henrich and Holmes [37] have shown concerns over the safety of a novel vaccine exist to some degrees among general population Canadians. Pandemics are, after all, situations that require vaccine production processes that must incorporate expedited testing and approvals protocols which could thereby cause some apprehension among some members of the public who feel the vaccine was not tested sufficiently [57]. Nevertheless, the significant prevalence of such concerns among Metis participants is certainly a compelling finding and strongly suggests that other mediating factors also likely had a hand in influencing their decision-making process. Despite some variability in vaccine uptake between urban/rural/remote regions, Metis participants commonly identified with a collective, colonially-informed experience of the pandemic throughout all the focus groups. Indeed, the colonial legacy functioned as a lens through which the pandemic was interpreted, inscribing events with an additional layer of meaning-making that did not exist for general population Canadians. Much like contemporary mistrust of vaccines among Latinos and African Americans in the United States has been shown to be rooted in their historical relationships with health and government agencies [8-10], Metis participants’ own collective colonial experiences mediated their perceptions of the pandemic and the vaccine response.

For over 100 years, Aboriginal people have held suspicions about the health systems in Canada and have often suspected that they were unwitting subjects of medical experiments [58]. In fact, such suspicions have been confirmed as recently as 2013 when it was uncovered that the Canadian government was withholding food from First Nations populations as part of experiments on malnutrition [59]. Such evidence underscores a common question of what other experiments could they have been a part of without their knowledge or consent. Similar to other areas of the world where the development of medical systems served the interests of the colonizing power [60], the Canadian health system co-evolved alongside the country’s longstanding colonial policies directed at territorial dispossession, social marginalization, assimilation, and controlling Aboriginal lives. Consequently, healthcare environments in Canada have historically been places where Aboriginal people often face overt and institutionalized racism [61,62]. Medical institutions can thereby continue to be seen by Aboriginal people in the present day as symbolic of the colonial project within which its practices have been historically embedded [63].

For Metis focus group participants, vaccinated or not, the sense of vulnerability conveyed in feeling like a “guinea pig” was a logical extension of a mistrust rooted in, and nurtured by, the colonial legacy. This explains how being named as a priority group for the vaccine – a status that has been shown to increase likelihood of vaccination [3,54] – came to have dual function, both positively and negatively influencing the decisions of some participants. As was similarly noted in a recent study that is part of the same broader project as this one, Metis and First Nations study participants wondered why they were prioritized, and without finding satisfactory answers, they became suspicious of the intent and purpose behind the vaccine campaign and came to their own conclusions. In particular, they likewise believed their lives were less valued in the eyes of the government, and rationalized that that they were being prioritized in order to test the safety of the vaccine before it was more broadly distributed [14].

In a similar fashion, the disproportionate rate of knowledge factors among Metis participants compared to general population Canadians in Boerner et al.’s study also directs attention towards the influence of related historical and contextual elements. Knowledge deficits about vaccines in general and the H1N1 vaccine in particular have also been expressed by other populations who have experienced histories of racism and social exclusion, including Latino and African Americans communities in the United States, as well as some indigenous Pacific Islanders [6-12]. Their prevalence and negative influence on the decision to vaccinate seems to confirm a link between the lower educational and socio-economic status (themselves being downstream manifestations of the social determinants of colonialism, racism, and social exclusion) [28], and lower health literacy and vaccine refusal [12,30]. These deficits can then contribute to increased concerns about vaccine safety as knowledge gaps are easily backfilled with fears, misconceptions, and myths that are ready to fill the void and lend a sense of opportune certainty in an otherwise uncertain time.

## Conclusion

Metis focus group participants commonly expressed that they felt starved of reliable information, and that health systems had left much of their informational needs unmet. In this case, Metis participants came to augment and amplify similar desires for more detailed information about the virus and the vaccine that have been found among other Canadians [64]. While one may be tempted into suggesting that the answer lies just in supplying the required information, caution should be urged against simply applying ‘deficit-model’ solutions [38]. Indeed, many participants’ self-acknowledged lack of vaccine/pandemic information complicates any facile notion of knowledge deficits by shedding light on the number of challenges that effective risk messaging faced – whether the obstacles were tied to the accessibility of messaging (in terms of both plain language availability as well as being broadly comprehensible), competition from common misconceptions, or the influence of socio-historical factors.

Recent risk communication research has emphasized the empowerment of the intended audience as an important goal of risk communication, rather than merely supplying information and in turn expecting compliance with vaccination recommendations – although the pressures of a public health crisis may heighten the tension between the two approaches [65-67]. In fact, overly simplistic ‘deficit-model’ approaches may actually reproduce colonial dynamics of hierarchy and paternalism and thereby decrease trust between communities and health systems [68]. Of course, people should receive information they need and want, but empowerment means that they also need to be able to understand the information, to feel engaged, and to have a sense of control, input, and meaningful participation in the risk dialogue. Empowerment is advancing the self-efficacy and decision-making capacity of the stakeholder in the ability to confidently take action (in whatever form that action ultimately takes) [69]. With this definition in mind, this study highlights the many instances in which the participants felt the exact opposite of feeling empowered.

What is more, empowerment should be a priority in a context where members of a targeted audience have experienced historic marginalization in a colonial setting [69]. Disparities in power differentials are characteristic to the colonial relationship, and incorporating a goal of empowerment into risk communication can help to infuse social justice imperatives into risk management activities [69], and, more broadly, assist the process of addressing historical wrongs and of decolonization. Different populations will interpret information in different and unique ways that reflect their cultural and social lives, and the results here demonstrate the importance of incorporating an overall ethical approach to risk communication. However, even when attempted, the success of highly tailored and community-derived interventions may be compromised if communications are delayed or face potential logistical challenges [70]. Clearly, balancing the ethical and logistical imperatives of risk communications can and will present difficulties, yet striving for their mutual improvement should not be considered a goal that is too impractical, or an ideal where one can only be effectively done at the expense of the other.

Nonetheless, with this ethical orientation in mind, it is fortunate that addressing knowledge gaps and dispelling misconceptions about vaccine safety can be two sides of the same coin. Pandemic planning and response measures should ensure that particular segments of the public have the information they need for sound and empowering decision-making, and in the process of which many myths could be simultaneously dispelled. Such efforts may increase the potential to raise vaccine uptake rates in a future pandemic scenario [56]. Despite negative perceptions attached to health systems among study participants, healthcare professionals are often a preferred information source [64], and their input had a substantial positive influence on vaccine uptake among participants in this study. Those who serve Aboriginal populations can build and leverage their role as a trusted information source to use the provider/patient interaction as an opportunity to listen to individuals’ concerns, as well as to discuss, inform, and educate. However, wholly relying on this approach will not be sufficient as this study showed that there may be other health care access issues at play, notably for Metis participants in the urban center of Winnipeg. Substantiating this likelihood is a recent report from the Métis Nation British Columbia (MNBC) – the Canadian province of British Columbia’s counterpart to the MMF – which indicates that many Métis people in that province face difficulties in accessing a family doctor [71].

The same MNBC report emphasized that social and support networks or community events are suitable mechanisms to share information and have health-related conversations with Métis citizens living in British Columbia. Likewise, some participants in this study suggested that they would liked to have had timely public forums or meetings held during the pandemic where they could offer their own input and find out the answers to the pressing questions and concerns that they had. They ideally wanted meetings with someone they trust in their communities who could be knowledgeable in pandemic issues, such as qualified local health personnel or community leaders. Public informational forums were not unknown in other health jurisdictions in Canada and the United States [72,73]. These could be further refined by: targeting populations with more specific and pressing information needs, incorporating collaborative and empowering pandemic planning and communication strategies, tailoring messaging to reflect and respect the audience’s culture and history, and accounting for inherent systemic barriers—such as access to a forum itself [39,74,75]. In fact, when reflecting on the H1N1 pandemic response, similarly-styled community information sessions were also suggested by members of remote First Nations communities in Canada. They believed that such forums, even held inter-pandemically, could help to prepare community members for future public health risks and pandemic scenarios and also help educate about vaccinations [76]. These suggestions could have significant potential to help establish a better dialogue between health systems and community members, while concurrently addressing misconceptions, providing greater assurances of vaccine safety, and informing about vaccine testing and approval processes [64].

The decision-making process can be complex and multifaceted for anyone considering a novel vaccine, and for Metis people it cannot be understood without also acknowledging the broader meta-context of colonialism that shaped their perceptions of risk and influenced their vaccine decisions. Recognizing such underlying factors and how they can mediate perceptions towards a vaccine can help health system risk communicators design more appropriate, effective, and empowering pandemic response strategies. Developing these kinds of strategies will be necessary, because despite a relatively high rate of vaccination among Metis participants, there was considerable apprehension among those who were vaccinated to take a similar vaccine in the event of a future pandemic. Along with the widespread fear of the vaccine, many participants expressed the opinion that the pandemic was “*blown out of proportion*” by media and health authorities since it was not seen to have been as threatening as initially thought – a sentiment that could decrease perceptions of vulnerability during a future pandemic scenario [77]. Added to this, there is the established presence of the anti-vaccination community on the internet, the medium where more and more people are looking for vaccine-related information [78]. Of course, a future pandemic could be much more severe, and in such a case that threat may trump vaccine safety concerns to even greater degrees than what occurred for many participants in this study. However, despite the classification of the H1N1 pandemic’s impact as moderate [79], its disproportionate impact on Aboriginal populations shows how improving vaccination campaigns to better reflect the realities and needs of the communities most at-risk is a valuable and potentially life-saving endeavor. At the same time, continuing to seek more thorough understandings of vaccine-related attitudes and perspectives is also a valuable part of these efforts, recognizing that beliefs evolve alongside respective influencers, such as ongoing colonial legacies, anti-vaccine movements, and the specter of new disease outbreaks.

## Abbreviations

SEM: Social-ecological model
MMF: Manitoba Metis Federation
MNBC: Métis Nation British Columbia
